# α-Hederin Inhibits the Proliferation of Hepatocellular Carcinoma Cells *via* Hippo-Yes-Associated Protein Signaling Pathway

**DOI:** 10.3389/fonc.2022.839603

**Published:** 2022-03-03

**Authors:** Tongqing Chen, Dongdong Sun, Qijuan Wang, Tingting Zhou, Jiani Tan, Changliang Xu, Haibo Cheng, Weixing Shen

**Affiliations:** Jiangsu Collaborative Innovation Center of Traditional Chinese Medicine Prevention and Treatment of Tumor, The First Clinical Medical College of Nanjing University of Chinese Medicine, Jiangsu, China

**Keywords:** α-hederin, hepatocellular carcinoma (HCC), Hippo signaling pathway, YAP protein, nuclear translocation

## Abstract

**Aims:**

Yes-associated protein (YAP), a downstream protein in the Hippo signaling pathway, plays an important role in tumor proliferation, including in hepatocellular carcinoma (HCC). α-hederin, a monodesmosidic triterpenoid saponin isolated from *Fructus akebiae*, displayed anti-cancer effects on several cancer cell lines but the precise mechanism has not been ascertained. In the present study, we explored the effects of α-hederin on cell proliferation and apoptosis in human HCC cell lines and the underlying mechanisms.

**Main Method:**

Cell proliferation and apoptosis were assessed using 5-ethynyl-2’-deoxyuridine staining, colony formation, flow cytometry. The expression patterns of components of Hippo signaling pathway and apoptotic genes were further examined *via* RT-qPCR and immunoblotting. A xenograft tumor model in nude mice was used to evaluate the anti-HCC effects of α-hederin *in vivo*.

**Results:**

α-hederin promoted the apoptosis and inhibited the proliferation of SMMC-7721 and HepG2 cells *in vitro*, and remarkably inhibited the tumor size and weight in the xenograft mouse model. Additionally, α-hederin increased the expression of pro-apoptosis proteins and suppressed the expression of anti-apoptosis proteins. Moreover, α-hederin treatment upregulated the expression of Hippo signaling pathway-related proteins and genes, while, effectively reduced the level of nuclear YAP, which resulted in the inhibition of proliferation and the induction of apoptosis of HCC cells. Finally, the effects of α-hederin on HCC cell proliferation and apoptosis were alleviated by XMU-MP-1, a Mst1/2 inhibitor *in vitro*.

**Significance:**

We identified α-hederin is a novel agonist of Hippo signaling pathway and possesses an anti-HCC efficacy through inhibiting YAP activity.

## Introduction

Liver cancer is one of the most malignant cancers with poor prognosis which already be the third leading cause of cancer-related death worldwide. Hepatocellular carcinoma (HCC) is the most common type of primary liver cancer, and its incidence and mortality keeps increasing (1). The higher recurrence and metastasis are responsible for the poor outcome (2). Although significant progress achieved from current basic and clinical investigations, our understanding of the tumorigenesis mechanisms in HCC is remained elusive. The complex pathology of HCC has limited the development of effective therapeutic intervention, prompting people to devote understanding of the tumorigenesis mechanisms and the therapeutic strategy discovery, including the search for effective substances in natural small molecule compounds for the treatment of HCC.

Recent studies have revealed that several developmental pathways, such as Hippo/Yes-associated protein (YAP) signaling, contribute to hepatic carcinogenesis (3). The Hippo signaling pathway was originally identified in Drosophila melanogaster and later in mammals (4, 5). It is an evolutionarily-conserved signaling pathway that plays an important role in organ size control, tissue regeneration, as well as tumor suppression (6). The core molecules of Hippo signaling pathway are serine/threonine kinases, mammalian sterile 20-like kinase 1/2 (Mst1/2), and large tumor suppressor 1/2 (Lats1/2), Mst1/2 kinases phosphorylate and activate Lats1/2, which in turn phosphorylates two transcriptional co-activators, YAP and WW domain-containing transcription regulator 1 (TAZ), contributing to their cytoplasmic sequestration and functional suppression (7–10). Moreover, accumulating evidence shows that dysregulation of the Hippo pathway is associated with a broad spectrum of cancers, such as liver cancer (11), breast cancer (12), non-small cell lung cancer (13), colon cancer (14).

α-hederin, an oleanane-type saponin is present in many plants. Several studies have proposed that α-hederin had an anti-cancer activity. For example, it inhibited interleukin 6–induced epithelial–mesenchymal transition in colon cancer cells and induced apoptosis in non-small cell lung cancer by increasing the killing effect of Tax (15, 16). Recently, researchers reported that α-hederin could induce apoptosis of HCC cells *via* the mitochondrial pathway mediated by increased intracellular ROS (17). However, the molecular mechanisms of α-hederin in anti-HCC progression are not fully understood. Based on the pretesting research, we found α-hederin could induce cell death in several hepatoma cells, so we speculate that Hippo signaling pathway might be involved in this mechanism. In the present study, we evaluated the effect of α-hederin on HCC proliferation in vivo and in vitro and explored the underlying molecular mechanism through investigating Hippo-YAP signaling pathway.

## Materials and Methods

All methods were performed in accordance with the relevant guidelines and regulations of our institution.

### Cell Culture and Reagents

The human SMMC-7721, HepG2, and Huh-7 HCC cell lines were purchased from ATCC. The cells were cultured in Dulbecco’s Minimum Essential Medium (DMEM) (Gibco, USA) with 10% FBS (Foetal Bovine Serum, BI), 100 U/mL penicillin, and 100 U/mL streptomycin at 37°C in a humidified atmosphere with 5% CO_2_. α-hederin was purchased from Chengdu Herbpurify CO., LTD (purity>98%). Dimethyl sulfoxide (DMSO) was used as solvent.

### Cell Viability Assay

Cells were seeded at a density of 8×10^3^ cells per well in 96-well plate and treated with 0, 2.5, 5, 10, 20, 40, 80 uM of α-hederin for 12 h, 24 h or 48 h followed by incubating with MTT solution for 4 h. The absorbance of each well was measured at 490 nm using the plate reader (TECAN SPARK 10M). Cell proliferation was assessed using MTT according to the manufacturer’s protocol (Beyotime, China).

### Western Blot

Antibodies: YAP (ab56701, abcam, 1:5000), Lats1 (ab70561, abcam,1:5000), p-YAP (ab62751, abcam,1:1000), Bax (ab32503, abcam,1:5000), Bcl-2 (ab196495, abcam,1:1000), TAZ (ab224239, abcam,1:5000), Caspase-3 (ab13847, abcam,1:500), GAPDH (ImmunoWay, YM3029,1:5000), Mst1 (CST,3682s,1:1000), TEAD1 (ab133533, abcam,1:5000), Cleaved-caspase3 (CST,9664s,1:1000), Phospho-Lats1/Lats2 (PA5-64591,Invitrogen,1:1000), Histone H3 (ImmunoWay, YM3038,1:5000).

Briefly, protein concentrations of tissues or cells were quantified by BCA protein assay kit (Thermo Scientific, Waltham, MA, USA). Sample of proteins (25 μg) were separated by 10% sodium dodecyl sulfate-polyacrylamide gel and transferred to PVDF membrane, then blocked by 5% non-fat milk in PBST buffer (PBS buffer containing 0.05% Triton-100) for 1 h at room temperature. The membranes were incubated overnight with primary antibodies at 4°C, after 3 times washing in PBST buffer, the membranes were incubated with the secondary antibodies for 1 h at room temperature. Signals were detected by using an ECL substrate (Thermo Scientific) and exposure with the Tanon 5500 imaging system. The intensity of the bands was quantified by densitometry.

### Immunofluorescence Assays

After drug treatment, cells were gently washed by PBS for three times and fixed with 4% fresh paraformaldehyde-phosphate (BL539A, Biosharp) for 15 min, then permeabilized with 0.5% Triton X-100 in PBS for 10 min at room temperature. After blocking in 10% goat serum and 5% BSA in PBS-T for 30 min, cells were incubated with primary antibody against YAP (1: 100) in 5% BSA overnight at 4°C. After washing three times with PBS-T, cells were incubated with secondary antibodies (1:200 dilution) for 1 h at room temperature. The coverslips were treated with DAPI for 5 min for nuclear staining. An inverted fluorescence microscope (Nikon Eclipse Ti, Nikon, Japan) was used for imaging.

### Flow Cytometry Analysis for Cell Cycle and Apoptosis

Apoptosis assay was monitored by flow cytometry. Cells were seeded at a density of 3 ×10^5/^ml in six-well plates. After attachment, cells were treated with α-hederin at different concentrations for 24 h. Supernatants of the cultures were collected and the attached cells were collected by digestion with pancreatin (S330JV, BasalMedia) followed by centrifugation (2000 rpm, 2min) after washing twice with PBS. Cells were re-suspended in 500 ul of 1×Binding Buffer and incubated with 5 ul of Annexin V-FITC and 5 ul of PI Staining Solution for 15 min. The next steps were followed according to the manufacturer’s instructions (Key GEN BioTECH).

For cell cycle analysis, cells were synchronized at the G0/G1 phase by serum starvation for 24 h and then released into cell cycle by re-addition of 10% FBS. After α-hederin treatment at different concentrations for 24 h, cells were collected and fixed in 75% ethanol at −20°C overnight, then re-suspended in precooled PBS for 3 times. Cell cycles were detected by the flow cytometer (Beckman Coulter) at 488 nm excitation wavelength and the data were analyzed with FlowJo/Modfit 5 software.

### 5-ethynyl-2’-Deoxyuridine Staining

To assess *in vitro* proliferation, the cells were supplemented with fresh medium containing 10 μM EdU and incubated for 2 h at room temperature after α-hederin treatment at different concentrations. Cell nuclei were counterstained with DAPI at room temperature for 10 min. EdU-positive cells were quantified using fluorescence microscopy (Nikon Eclipse Ti, Nikon, Japan).

### Colony Formation Assay

Cells were seeded in six-well plates and treated with different concentrations of α-hederin for 24 h. Then, the medium was replaced with α-hederin free medium and continued to incubate for about 14 days. The cells were fixed with 4% paraformaldehyde and stained with Giemsa stain at room temperature. After a final series of rinses and air dry, photographs were taken for colony quantification.

### RT- qPCR Analysis

Total RNA was extracted using the Trizol reagent (Invitrogen, Carlsbad, CA) according to the manufacturer instructions. 1 μg of total RNA was reversed transcription to cDNA by using the *Evo M-MLV* RT Mix Kit with gDNA Clean for qPCR (AG11728, ACCURATE BIOTECHNOLOGY, HUNAN, Co.,Ltd). The qPCR was performed using the SYBR Green Premix *Pro Taq* HS qPCR Kit (AG11701, ACCURATE BIOTECHNOLOGY, HUNAN, Co., Ltd). The expression level of individual genes was analyzed by the comparative Ct method (2^-ΔΔCt^ method) and normalized according to the expression of the housekeeping gene.

### Animal Experiments

All animal experiments were approved by our Animal Ethics Committee of Nanjing University of Chinese Medicine (Nanjing, China), and all experiments were performed in accordance with relevant guidelines and regulations. For each mouse, 0.2 ml (2.5×10^7^/ml) HepG2 cell suspension was injected subcutaneously into the right axillary region of female BALB/c nude mice (6 weeks). All animals were maintained in a pathogen-free and temperature-controlled environment with 12 h light/dark cycle and standard laboratory diet. The animals were randomized into three groups (n=8 per group): control group, α-hederin group (5 mg/kg), and positive group DDP (Cisplatin, 5 mg/kg). Tumor growth was calculated with calipers every three days, and the volume was calculated according to the formula: volume = (width)^2^ × length/2. The body weight of the mice was recorded every three days. At the end of experiments, xenotransplant tumors (three mice per group were chosen randomly to be identified by western blot), livers, mouse blood and kidney were harvested for additional analysis.

### Live Animal Imaging

For fluorescence imaging *in vivo*, mice were imaged using an IVIS Spectrum *In Vivo* Imaging System (Caliper Life Sciences). HepG2 cells were transfected with a green fluorescent protein (GFP) vector [Zhongqiao Xinzhou Science and Technology Co. (Shanghai, China)]. Prior to *in-vivo* imaging, the mice were anesthetized with isoflurane. Excitation of fluorophore were performed at 488 nm for GFP. The signals were analyzed with Living Image Software (PerkinElmer).

### Hematoxylin-Eosin Staining

The xenograft tumor tissues from different groups of mice were fixed in 4% paraformaldehyde for over 12 h and were embedded in paraffin. Then, the tissues were cut into 4 μm thick sections and stained with hematoxylin and eosin (HE).

### Immunohistochemistry Staining

For Immunohistochemistry, paraffin sections were incubated with a blocking solution and then incubated with anti-YAP antibody (Abcam, ab52771) at 4°C overnight. After washing, sections were incubated with biotinylated secondary antibodies at room temperature for 30 min. Sections were visualized with 3, 3’-diaminobenzidine and then counterstained and dehydrated for microscopic observation (×100, ×200, Nikon, Japan).

### TUNEL Staining Assay

The One Step TUNEL Apoptosis Assay Kit (Beyotime) was used for TUNEL assay. The 4um-thick paraffin‐embedded tissue sections were dewaxed with xylene and rehydrated in an ethanol gradient. Then, the slices were treated with 20 ug/ml proteinase K for 30min at 37°C. After washed three times with PBS for 5min, the slices were incubated with TUNEL reaction mixture for 1 h at 37°C away from light, washed three times in PBS, then incubated with DAPI-containing anti-fluorescence quenching tablets and observed under a fluorescence microscope.

### Statistical Analysis

All data are presented as the mean ± standard deviation (SD) of at least three independent experiments. Student’s t-test determined statistical differences between samples and the Bonferroni *post hoc* procedure was performed for a one-way analysis of variance (ANOVA) of statistical comparisons between more than two samples. GraphPad Prism 7.0 (GraphPad, La Jolla, CA, USA) was used to create the resulting data charts. Statistical significance was considered to be p < 0.05 or p < 0.01.

## Results

### α-Hederin Inhibited the Proliferation of HCC Cell Lines

**Figure 1:**
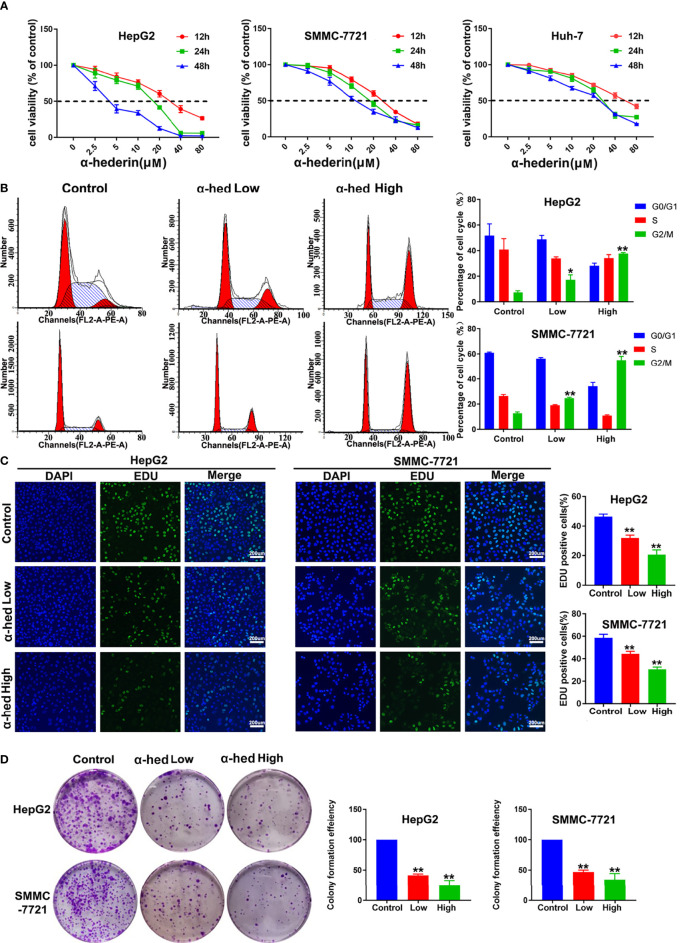
α-hederin inhibited the proliferation of HCC cell lines. **(A)** HepG2, SMMC-7721 or Huh-7 cells were treated with the indicated concentrations of α-hederin for 12, 24 or 48 hours, followed by MTT cell viability assay. **(B)** Cell cycle analysis of HepG2 and SMMC-7721 cells treated with α-hederin at low (10 µM) or high (20 µM) concentration for 24 hours. **(C)** Representative images and analysis of EdU staining in HepG2 and SMMC-7721 cells treated with α-hederin at low (10 µM) or high (20 µM) concentration for 24 hours. **(D)** Representative images and quantitative analysis of colony numbers of HepG2 and SMMC-7721 cells treated with α-hederin at low (10 µM) or high (20 µM) concentration for 24 hours. Significance: *p < 0.05 versus control, **p < 0.01 versus control.

To investigate the effects of α-hederin on HCC cells proliferation, we treated HCC cell lines with indicated concentrations of α-hederin for 12 h, 24 h, and 48 h. Notably, α-hederin inhibited the growth of HCC cell lines in a time and dose-dependent manner (**Figure 1A**). The IC50 values of HepG2, SMMC-7721, Huh-7 cells at 24 h were 18.5 µM, 17.72 µM, 21.89 µM, respectively. Accordingly, the treatment of α-hederin at 10 µM (low concentration) and 20 µM (high concentration)for 24 h was selected for subsequent experiments. By Flow cytometry, we found thatα-hederin significantly promoted the accumulation of the G2/M phase compared with the control group (**Figure 1B**). In addition, the EdU assay revealed a marked reduction of the proliferative ability of HepG2 and SMMC-7721 cells after being treated with low and high concentrations of α-hederin (**Figure 1C**).

To further confirm the inhibitory effect of α-hederin on hepatoma cell proliferation, cell clone formation experiment was performed (**Figure 1D**). Our results clearly displayed that α-hederin inhibited the colony-formation at the indicated concentration. The results described above indicate that α-hederin exerted an inhibitory effect on the proliferation of HCC cells.

### α-Hederin Promoted Apoptosis of HCC Cell Lines *In Vitro*

**Figure 2:**
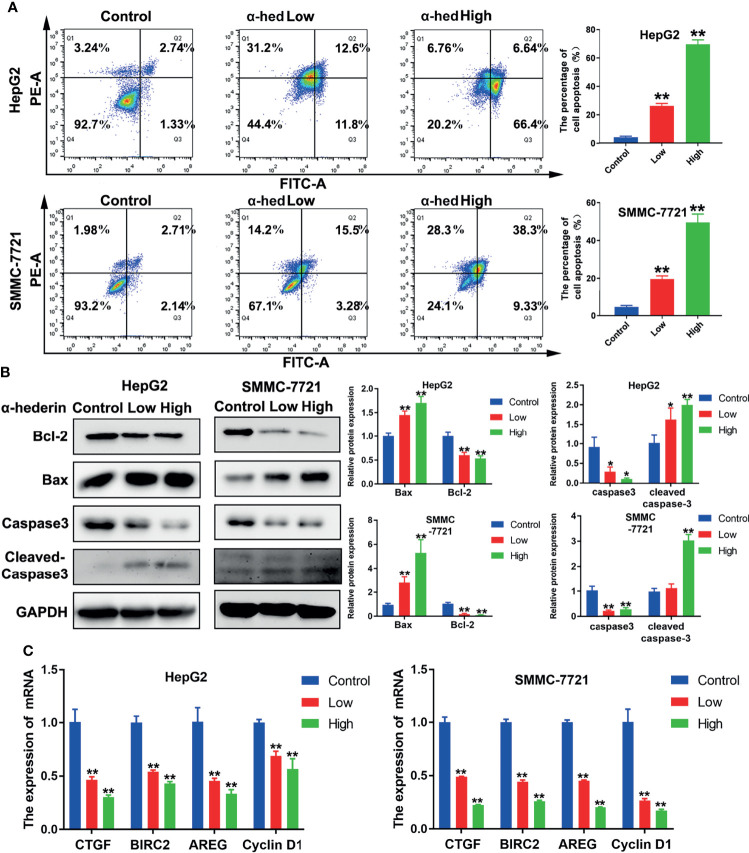
α-hederin promoted apoptosis of HCC cell lines *in vitro*. **(A)** Analysis of apoptosis in HepG2 and SMMC-7721 cells treated with α-hederin for 24 hours by flow cytometry. **(B)** The protein levels of Bax, Bcl-2, caspase 3, and cleaved caspase 3 were detected by Western blot in HepG2 and SMMC-7721 cells treated with the indicated concentrations of α-hederin for 24 hours. **(C)** Analysis of genes associated with cell proliferation and apoptosis in HepG2 and SMMC-7721 cells treated with α-hederin for 24 hours. Significance: *p < 0.05; **p < 0.01 versus control.

Apoptosis of HCC cells induced by α-hederin was analyzed by flow cytometry and morphological observation. We found that α-hederin increased the proportion of apoptotic cells (**Figure 2A**). Compared with that of control cells, the proportion of apoptosis in HepG2 cells treated with 10 µM or 20 µM α-hederin increased from 3.95% to 26.13% and 69.58% respectively. Similar results were found in SMMC-7721 cells, the proportion of apoptotic cells increased from 4.85% to 18.78% and 54.7% respectively by 10 µM or 20 µM α-hederin treatment. The effect of apoptosis induced by α-hederin was verified at the level of protein expression. The apoptosis-related proteins were determined by Western blot. The results revealed that the expression of Bax and cleaved-caspase3 were obviously increased by α-hederin treatment, while the expression of Bcl-2 and caspase-3 in HepG2 and SMMC-7721 cells were significantly decreased in a dose-dependent manner (**Figure 2B**). Furthermore, the expression of genes associated with apoptosis and proliferation were measured. As shown in **Figure 2C**, the proliferation-associated genes including CTGF, BIRC2, AREG, and Cyclin D1 in HCC cells were significantly decreased by α-hederin treatment compared with the control cells.

### α-Hederin Inhibited YAP Activation *via* Upregulating Hippo- Signaling Pathway

**Figure 3:**
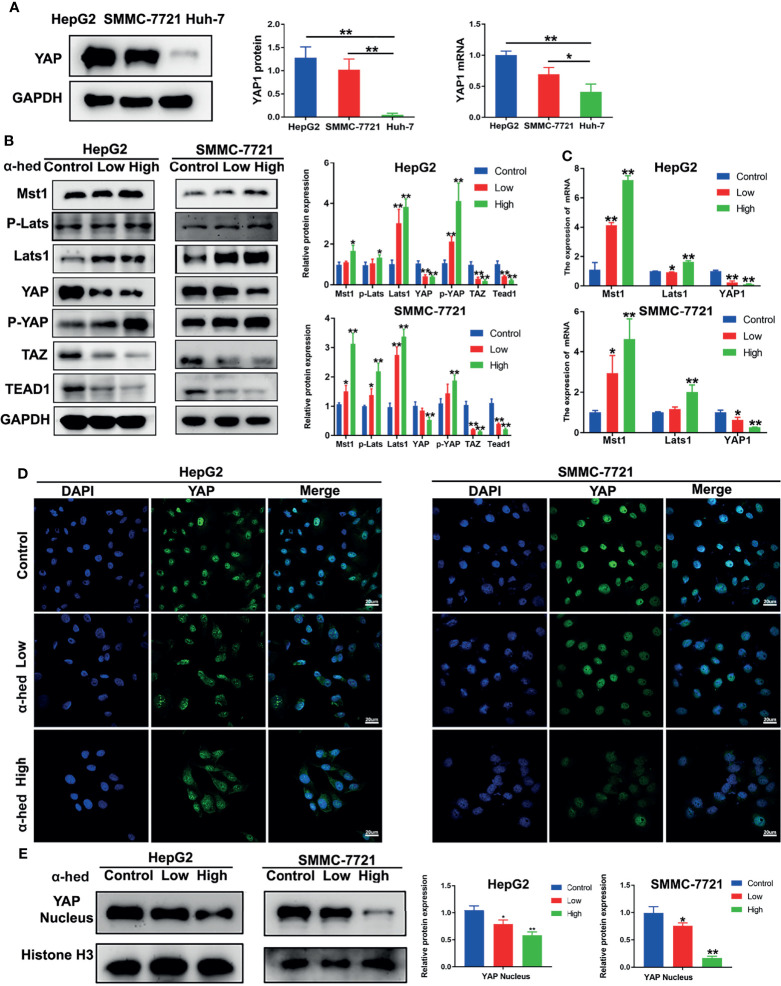
α-hederin inhibited YAP activation *via* upregulating the activation of Hippo signaling pathway. **(A)** The protein and mRNA and levels of YAP in HepG2, SMMC-7721, Huh-7 cells. **(B)** HepG2 and SMMC-7721 cells were treated with α-hederin at low (10 µM) or high (20 µM) concentration for 24 h, and then the proteins involved in Hippo-YAP signaling pathway were analyzed by Western blot, followed by densitometric quantification. **(C)** The mRNA levels of Mst1, Lats1, YAP in HepG2 and SMMC-7721 cells treated with α-hederin for 24 hours were measured by RT-qPCR. **(D)** Immunofluorescence analysis of YAP in HepG2 and SMMC-7721 cells. **(E)** The nuclear levels of YAP in HepG2 and SMMC-7721 cells treated with α-hederin at low (10 µM) or high (20 µM) were analyzed by Western blot. Significance: *p < 0.05 versus control, **p < 0.01 versus control.

YAP is a downstream protein of Hippo signaling pathway, and plays a key role in hepatic carcinogenesis. We firstly investigated the native expression of YAP at gene and protein level in Huh-7, HepG2, and SMMC-7721 cells. As shown in **Figure 3A**, both YAP gene and protein highly expressed in HepG2 and SMMC-7721 cells compared with Huh-7 cells. Therefore, in the present study, HepG2 cells and SMMC-7721 cells were used. Crucial molecules of Hippo signaling pathway in HepG2 and SMMC-7721 cells were further examined. We found that α-hederin treatment suppressed YAP/TAZ protein expression, but elevated the expression of Mst1, Lats1, P-Lats, P-YAP in a dose dependent manner. Accordingly, the level of TEAD1 transcription factor was downregulated (**Figure 3B**). RT-qPCR results revealed that α-hederin treatment effectively enhanced Mst1 and Lats1 gene expression while downregulated YAP gene expression in HepG2 and SMMC-7721 cells (**Figure 3C**).

YAP is inactivated by phosphorylation followed by degradation in the cytoplasm (7, 18, 19). To verify whether α-hederin could inhibit YAP signaling activity, fluorescence staining followed by confocal microscopy was used. Our results showed that in both HepG2 and SMMC-7721 cells, α-hederin treatment cells revealed a significantly decreased distribution of green fluorescence in the nucleus and an increased cytoplasmic staining in comparing with that in control cells (**Figure 3D**), and the reduced level of nuclear YAP was confirmed by immunoblotting (**Figure 3E**). These results suggested that the inhibitory effect of α-hederin on HCC cell proliferation may be due to the suppression of YAP activation caused by the upregulated Mst1 activation.

### Inhibition of Mst1/2 Activation Reversed the Inhibitory Effect of α-Hederin on Hepatoma Cell Proliferation

**Figure 4:**
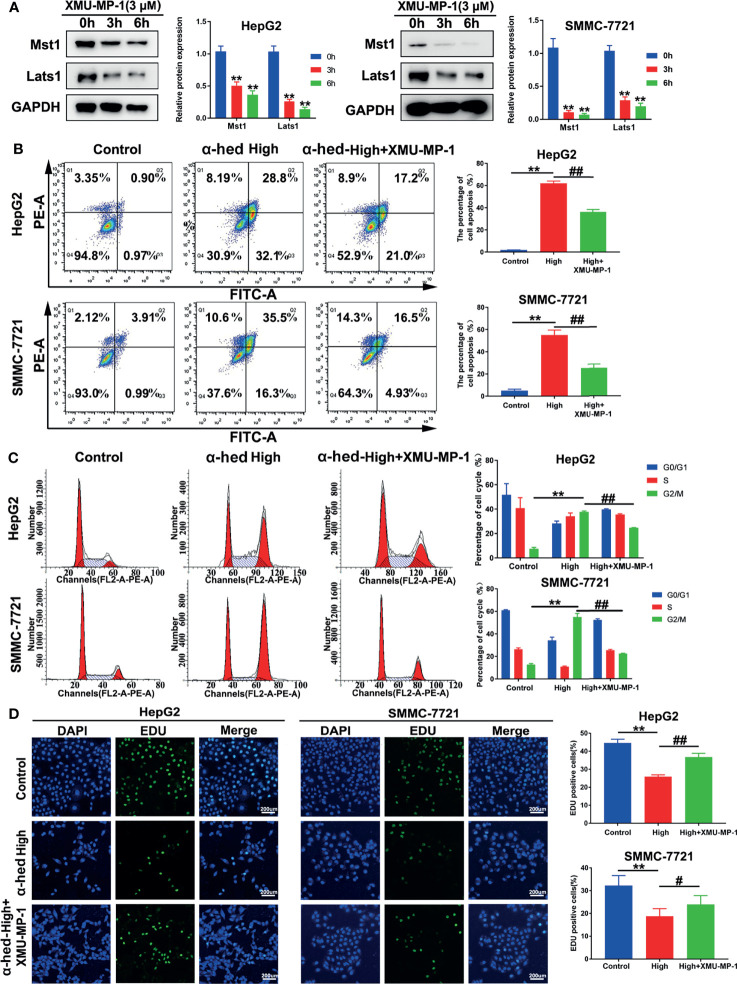
Inhibition of Mst1 activation reversed the effect of α-hederin on hepatoma cell proliferation. **(A)** Western blot analysis of Mst1 and Lats1 expression in HepG2 and SMMC-7721 cells treated with XMU-MP-1 for 3 h or 6 h. **(B)** Flow cytometric analysis of apoptotic HepG2 and SMMC-7721 cells after α-hederin or α-hederin/XMU-MP-1 treatment. **(C)** Flow cytometric analysis of G1- and G2/M-phase subpopulations in HepG2 and SMMC-7721 cells treated with α-hederin or α-hederin/XMU-MP-1. **(D)** Cell proliferation assay using EDU labeling in HepG2 and SMMC-7721 cells treated with α-hederin or α-hederin/XMU-MP-1. Significance: **p < 0.01 versus control, #p<0.05 versus High concentration, ##*p* < 0.01 versus High concentration.

To further verify the role of Mst1/2 in the suppression of hepatoma cell proliferation caused by α-hederin, HepG2 and SMMC-7721 cells were treated with Mst1/2 inhibitor XMU-MP-1 (3 μmol) for 3 h or 6 h before exposure to α-hederin. Immunoblot results revealed that XMU-MP-1 effectively inhibited the expression of Mst1 and downstream kinase Lats1 (**Figure 4A**). Additionally, XMU-MP-1 suppressed the α-hederin-induced reductions in cell density and the irregular morphology of HepG2 and SMMC-7721 cells. Moreover, the results of flow cytometric analysis showed XMU-MP-1 treatment not only ameliorated apoptosis, but also prevented cell cycle arrest induced by α-hederin (**Figures 4B, C**). Data from the EdU assay showed that XMU-MP-1 treatment reversed the inhibition of proliferation caused by α-hederin in HepG2 and SMMC-7721 cells (**Figure 4D**).

### α-Hederin-Induced Mst1 Upregulation Was Responsible for the Inhibition of YAP Signaling Pathway

**Figure 5:**
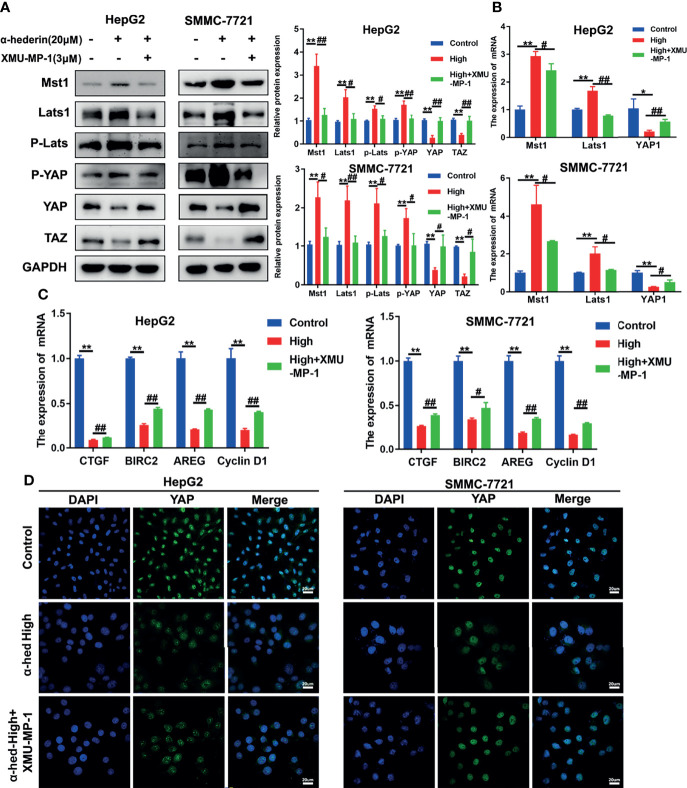
α-hederin-induced Mst1 upregulation was responsible to the inhibition of YAP signaling pathway. **(A)** The expression of proteins involved in Hippo-YAP signaling pathway in HepG2 and SMMC-7721 cells that treated with α-hederin or α-hederin/XMU-MP-1 was analyzed by Western blot. **(B)** The expression of Hippo-YAP signaling target genes in HepG2 and SMMC-7721 cells that treated with α-hederin or α-hederin/XMU-MP-1 was analyzed by RT-qPCR. **(C)** Analysis of genes associated with cell proliferation and apoptosis in HepG2 and SMMC-7721 cells treated with α-hederin or α-hederin/XMU-MP-1. **(D)** Representative immunofluorescence images of YAP in HepG2 and SMMC-7721 cells with the indicated treatment. Significance: *p < 0.05 versus control, **p < 0.01 versus control, #*p* < 0.05 versus High concentration, ##*p* < 0.01 versus High concentration.

To further validate whether the decreased YAP activation induced by α-hederin was due to the increased Mst 1/2 activity. HepG2 and SMMC-7721 cells were treated with α-hederin or a combination of α-hederin/XMU-MP-1 for 24 h. Immunoblot revealed that XMU-MP-1 treatment reversed the α-hederin-induced expression changes of Mst1, Lats1, P-Lats1/2, YAP, P-YAP, TAZ in HCC cells (**Figure 5A**). These results were also supported by RT-qPCR analysis (**Figure 5B**). Moreover, XMU-MP-1 could partially reverse the α-hederin-induced decrease of the mRNA expression of YAP target genes involved in proliferation and apoptosis(**Figure 5C**). Furthermore, the immunofluorescence analysis results showed that XMU-MP-1 treatment prevented the reduction of nuclear YAP induced by α-hederin (**Figure 5D**). Our results demonstrated that the Hippo signaling pathway played a critical role in α-hederin-medicated inhibition of cell proliferation and promotion of apoptosis in HCC cell lines.

### α-Hederin Attenuated Xenograft Tumor Growth of Human HCC *In Vivo*

**Figure 6:**
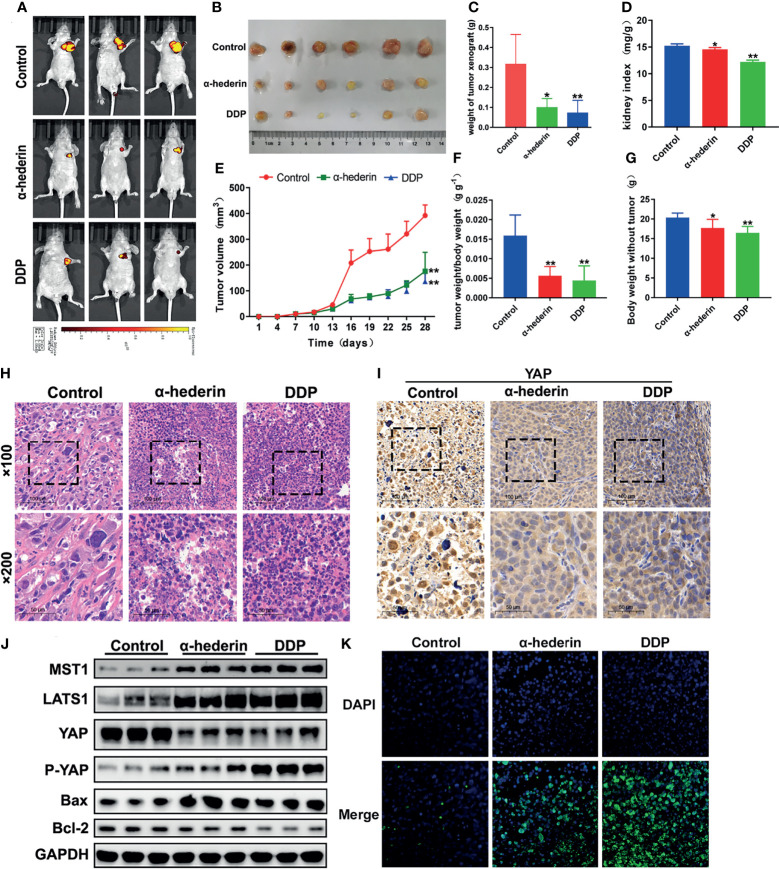
α-hederin attenuated xenograft HCC tumor growth *in vivo* through inhibiting YAP signaling pathway. **(A)** *In vivo* imaging of the HepG2 cell xenograft model mice. **(B)** Representative images of tumors collected from HepG2 cell xenograft model mice treated with PBS, α-hederin and DDP. **(C)** Weight of xenograft tumors harvested from mice. **(D)** Kidney index (kidney weight/bodyweight). **(E)** Tumor volumes were measured at different time points. **(F)** The ratio of tumor weight/body weight. **(G)** Mice body weight subtracted tumor at the end point. **(H)** HE staining of representative tumor tissues from different groups. **(I)** Immunohistochemistry analysis of YAP in mouse tumor tissues. **(J)** Immunoblot analysis of Hippo-YAP signaling pathway-related proteins and apoptosis-related proteins in the tumor tissues. **(K)** TUNEL staining of tumor tissues. Significance: *p < 0.05 versus control, **p < 0.01 versus control.

Although we have been already verified that α-hederin could inhibit the proliferation of human HCC cells and induce its apoptosis *in vitro*, but whether α-hederin could display the same effects *in vivo* should be investigated. HepG2 cells which are stably transformed with green fluorescent protein were injected into BALB/c nude mice, then treated with α-hederin or DDP as a positive control. *In-vivo* fluorescence imaging confirmed that the fluorescence intensity of HepG2 cells was significantly attenuated and the areas of fluorescence were restricted by α-hederin treatment (**Figure 6A**). Compared with the control mice, the α-hederin-treated mice exhibited a smaller tumor size and lower tumor weight (**Figures 6B, C, E**). Besides, the kidney index and bodyweight of mice in the α-hederin group were higher than in the DDP group, this means α-hederin has low side effects (**Figures 6D, F, G**). The sections of the HepG2 xenograft tumor were analyzed by HE staining (**Figure 6H**). Compared with the control group, neoplastic cells in α-hederin or DDP treatment group underwent cell death as evidenced by nuclear pyknosis and loss disintegration of cellular architecture, this result was consistent with restricted xenograft tumor size (**Figures 6B, C**) and induced apoptosis in **Figure 2**. The expression of YAP was analyzed in tumor sections (**Figure 6I**) and total proteins were extracted from tumor tissue (**Figure 6J**). After treatment with α-hederin and DDP, the expression of the apoptosis‐promoting protein (Bax) was upregulated, whereas the apoptosis‐inhibiting protein (Bcl-2) was downregulated. Besides, the main upstream Hippo pathway kinases (MST1, LATS1) were upregulated. More importantly, the expression of P-YAP was increased, whereas that of YAP was decreased after treatment with α-hederin and DDP. Besides, TUNEL staining assay showed more apoptosis cells in α-hederin and DDP group (**Figure 6K**).

## Discussion

In this study, we have identified that α-hederin as a potential suppressor in hepatocellular carcinoma *via* inhibiting YAP activity. Taking advantage of live animal imaging, we clearly showed that α-hederin exhibited a strong inhibitory effect on liver cancer growth and progression. Besides, our data showed that α-hederin increased the expression of Hippo signaling pathway proteins MST1, LATS1, P-LATS, and P-YAP in HCC. Moreover, the effects of above proteins expression could be reversed by XMU-MP-1, a reversible Mst1/2 inhibitor. Thus, our data provided a novel mechanism of α-hederin in inhibiting hepatocellular carcinoma through the Mst1/2-mediated activation of Hippo signaling pathway.

HCC is one of the most common types of liver cancer, with a global cancer mortality rate of 8.2% (20). Considerable progress in the understanding of HCC pathogenesis in recent years entails substantial advances in the diagnosis and therapy of that disease (21–23). Chinese herbs, such as evodiamine and limonin (24, 25), were reported to have alleviatory effects on HCC progression. Fruit of Fiverleaf Akebia, a traditional Chinese medicine, is widely used in the clinic. The active ingredient, α-hederin, has been confirmed to induce apoptosis in various human cancer cell lines.

The Hippo signaling pathway is the master regulator of organ development and also plays a critical role in live size control (26, 27). Besides, the Hippo signaling pathway has been implicated in cancer development. Both YAP and TAZ are key downstream effectors of the Hippo pathway and have been confirmed upregulated in a wide range of human cancer (28, 29). Upon activation, YAP and TAZ accumulate in the nucleus and then bind to the transcription factors such as TEAD, thereby regulate the expression of target genes that promote cell proliferation and cell survival. An earlier clinical study has confirmed that YAP expression was up-regulated in HCC patients (30). In our experiment, YAP overexpression was clearly observed in HepG2, SMMC-7721 cells, but not Huh-7 cells.

To our knowledge, there are no studies have identified the potential role of α-hederin in the Hippo signaling pathway in HCC cell lines. In the current study, we explored whether α-hederin exerted its anti-tumor activity by mediating Hippo signaling pathway both *in vivo* and vitro. The real-time PCR results revealed dramatic alterations in main components of Hippo signaling pathway. In HCC cell line, α-hederin treatment led to the significant inhibition of YAP expression through the upregulation of Mst and Lats phosphorylation, leading to phosphorylation and decreased nuclear translocation of YAP. The majority kinases of Hippo signaling pathway play a critical role in tumor suppression, and thus, present optimal small molecular targets. Therefore, inhibiting YAP/TAZ-TEAD by upregulating Hippo signaling pathway activity is an attractive and viable option for cancer therapy (31). Furthermore, development a small molecular agonist that could effectively restore the function of Mst1/2 or Lats1/2 kinases is a major challenge (32). XMU-MP-1 is a reversible and selective Mst1/2 inhibitor, could effectively inhibit Hippo signaling pathway (33). The function of XMU-MP-1 partially reversed phenotypes through suppressing the upregulation of Mst1/Lats1 and YAP phosphorylation induced by α-hederin. As we all know, Mst1 is an upstream kinase of YAP and also have other important cellular targets. Limited relevant research showed that Lats1/2 is still active and suppressed YAP activity in the absence of Mst1/2 (34). In that case, a future interest is to assess whether knockout Lats1/2 can directly reverse the effects of α-hederin treatment in HCC cell lines. Furthermore, relevant research found that YAP and TAZ in normal hepatocytes and tumor cells act through a competitive mechanism to eliminate tumor cells (35). Therefore, the specific mechanisms of Hippo signaling pathway remain to be elucidated. In summary, our study suggests that α-hederin exerted an anti-HCC effect through upregulating Hippo signaling pathway activation, which resulted in the inhibition of YAP activity. However, the further research is warranted to establish the precise mechanisms underlying the action of α-hederin along with validation in various animal models and clinical studies.

## Conclusion

Our results revealed that α-hederin acted as a new agonist of the Mst1-mediated Hippo signaling pathway and played an inhibitory role in hepatocellular carcinoma (HCC) growth through inhibiting YAP activity. The data obtained in this study provide an important base for further research into the utility of α-hederin as a potential therapeutic or preventive candidate agent for hepatocellular carcinoma (HCC) therapy.

## Data Availability Statement

The original contributions presented in the study are included in the article/**Supplementary Material**. Further inquiries can be directed to the corresponding authors.

## Ethics Statement

The animal study was reviewed and approved by the Animal Ethics Committee of Nanjing University of Chinese Medicine.

## Author Contributions

WS supported and supervised the study. TC, QW, and TZ performed the animal experimental assays. TC, JT, and CX analyzed data. TC, HC, and QW, conducted the study. TC wrote the manuscript. All authors contributed to the article and approved the submitted version.

## Funding

This work was supported by the National Natural Science Foundation of China (81973523, 81930117), A Project Funded by the Priority Academic Program Development of Jiangsu Higher Education Institutions.

## Conflict of Interest

The authors declare that the research was conducted in the absence of any commercial or financial relationships that could be construed as a potential conflict of interest.

## Publisher’s Note

All claims expressed in this article are solely those of the authors and do not necessarily represent those of their affiliated organizations, or those of the publisher, the editors and the reviewers. Any product that may be evaluated in this article, or claim that may be made by its manufacturer, is not guaranteed or endorsed by the publisher.
