# Supplementary material for: α-Hederin Inhibits the Proliferation of Hepatocellular Carcinoma Cells via Hippo-Yes-Associated Protein Signaling Pathway
Source: Front Oncol. 2022 Mar 3;12:839603. doi: 10.3389/fonc.2022.839603 (PMC8927085; doi:10.3389/fonc.2022.839603)

HepG2 （Figure 2）

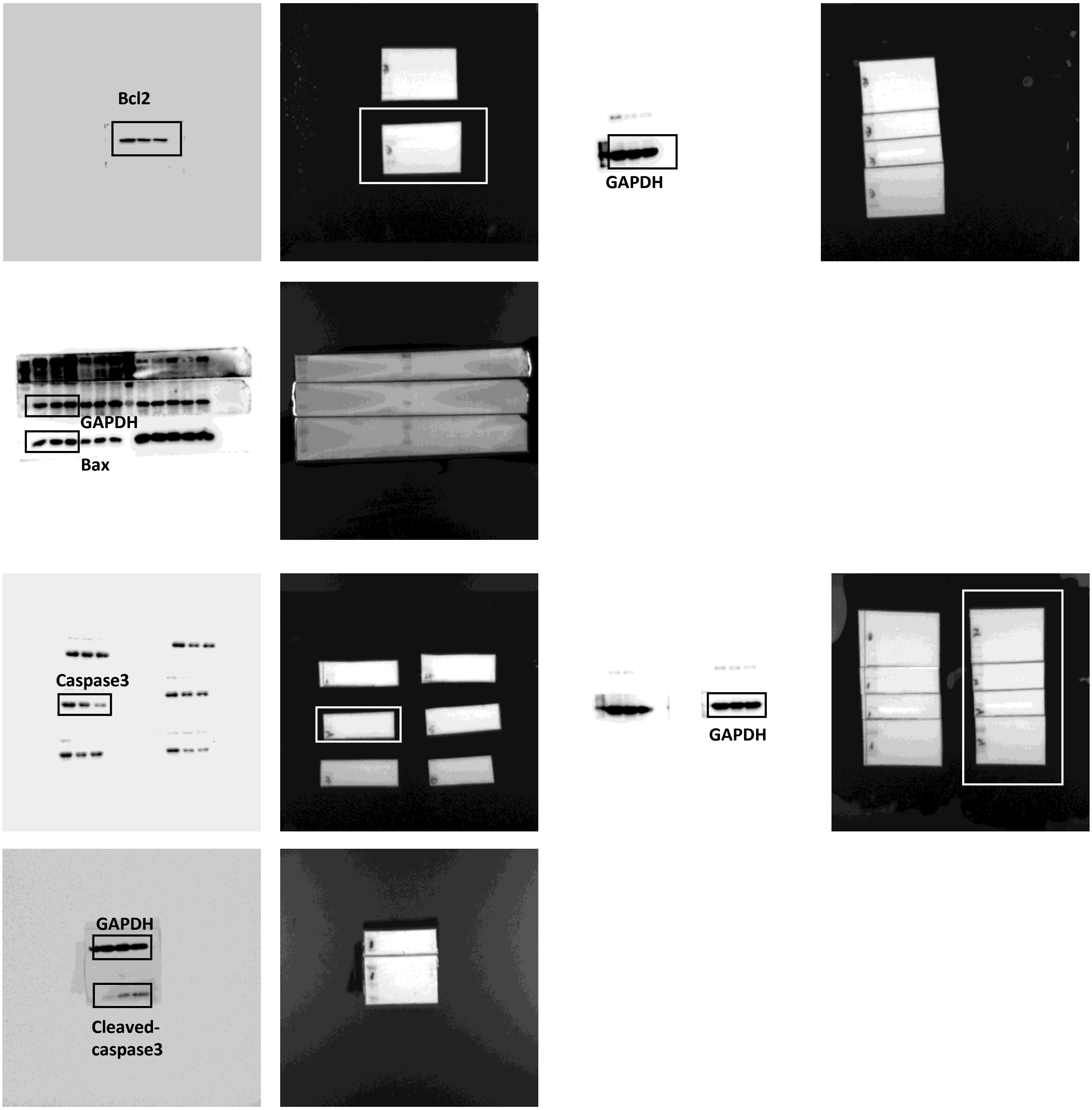

SMMC-7721 （Figure2）

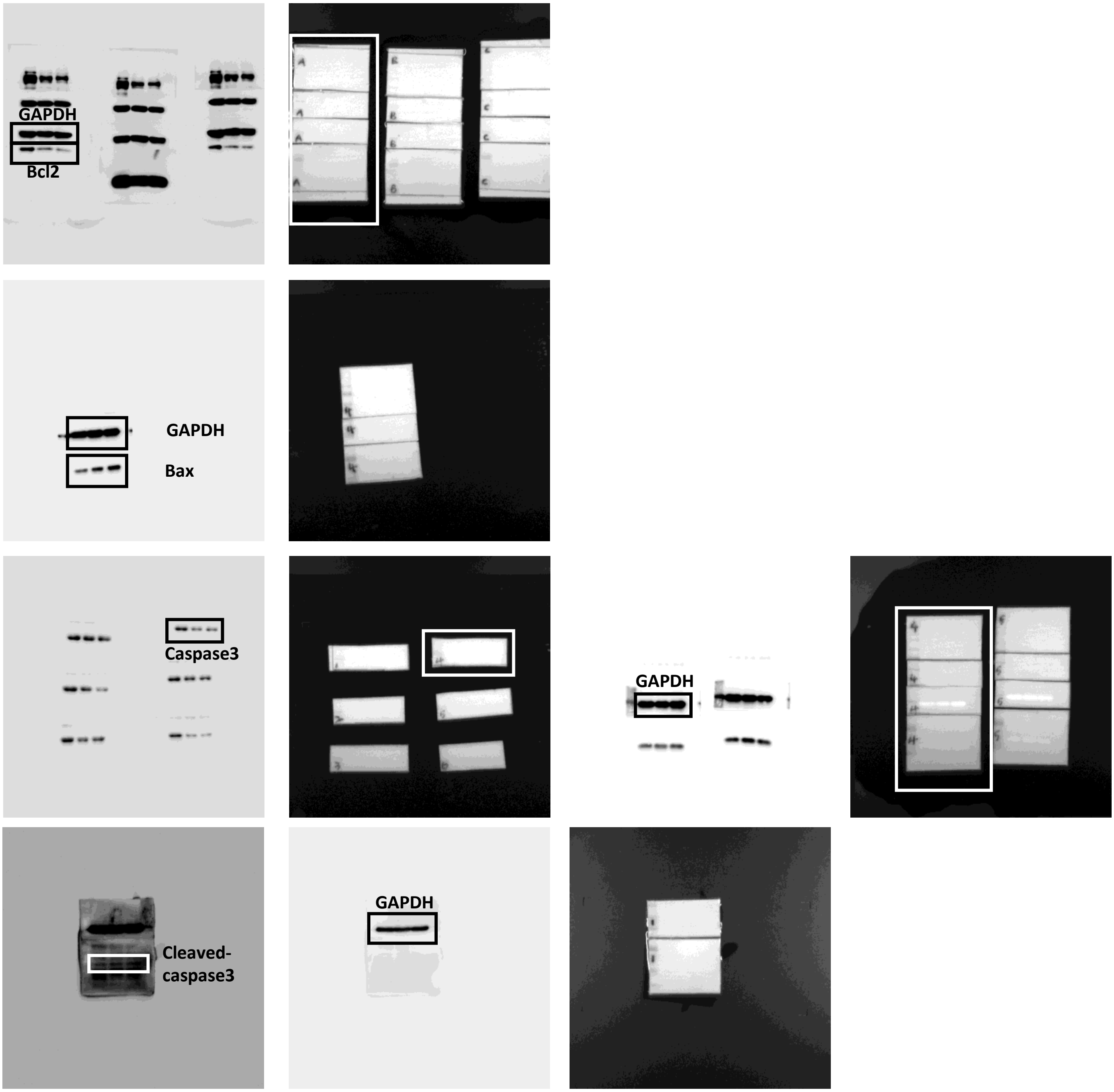

HepG2 （Figure 3）

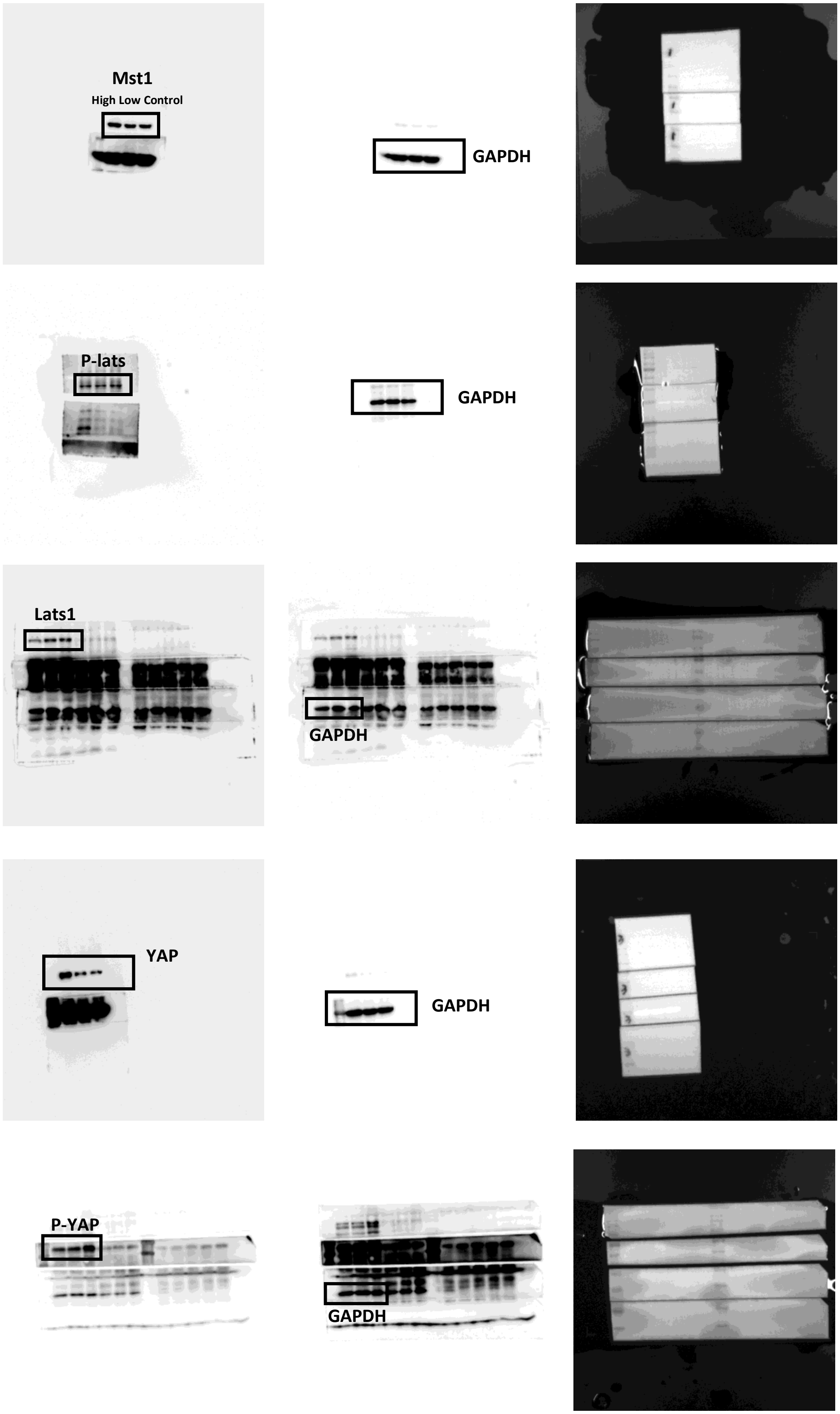

HepG2 （Figure 3）

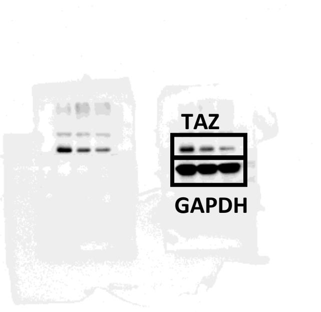

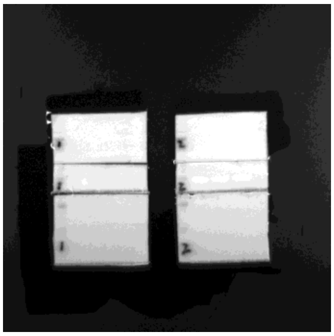

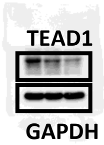

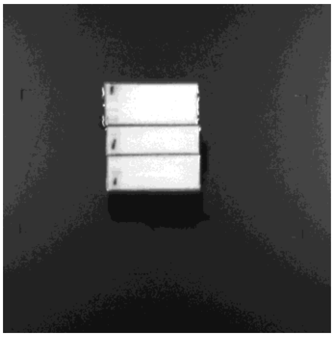

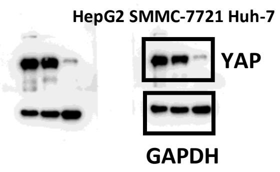

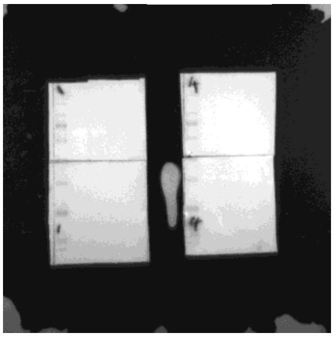

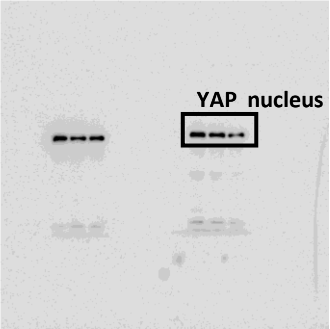

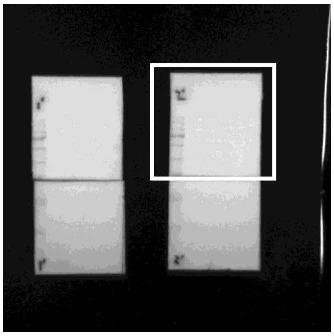

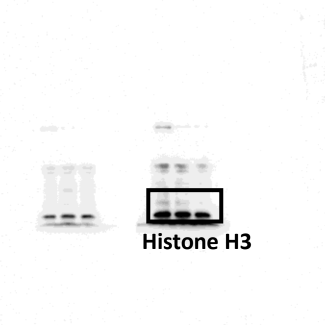

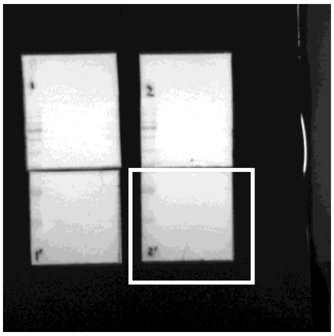

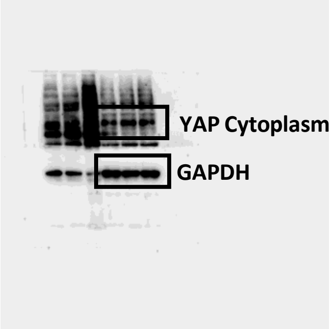

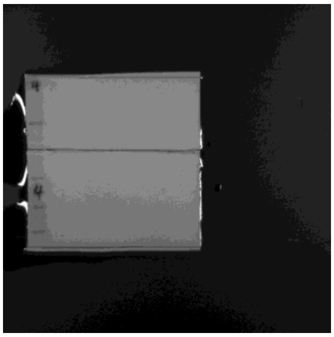

SMMC-7721 （Figure 3）

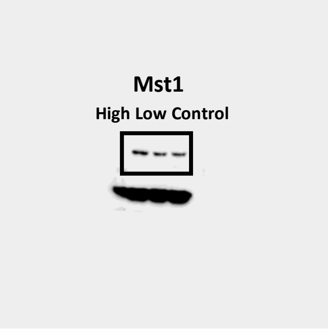

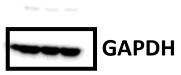

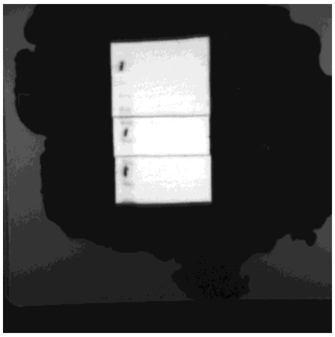

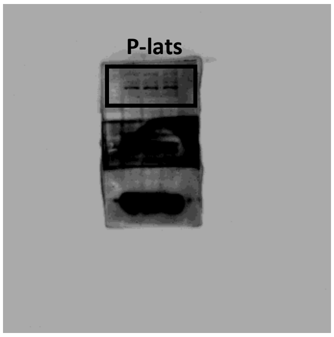

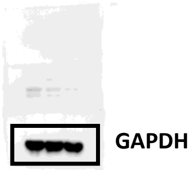

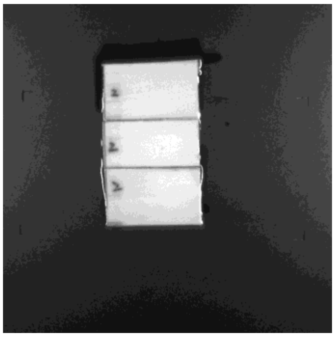

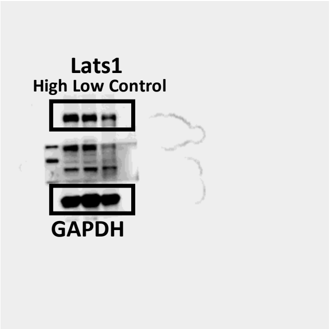

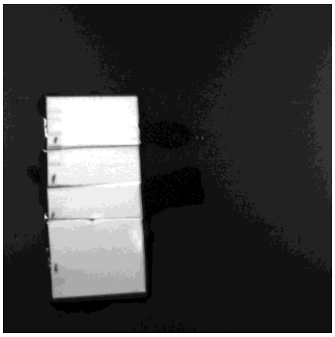

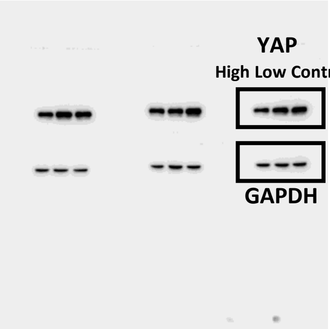

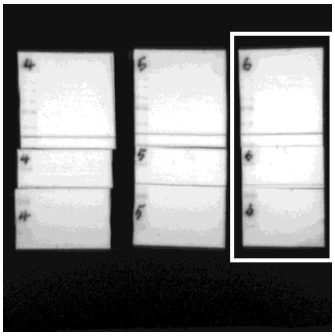

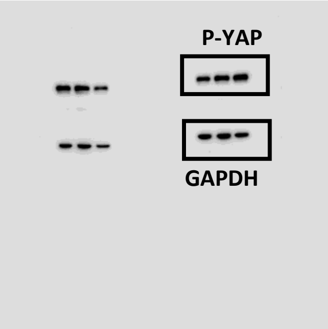

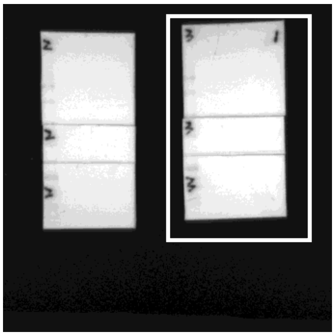

SMMC-7721 （Figure 3）

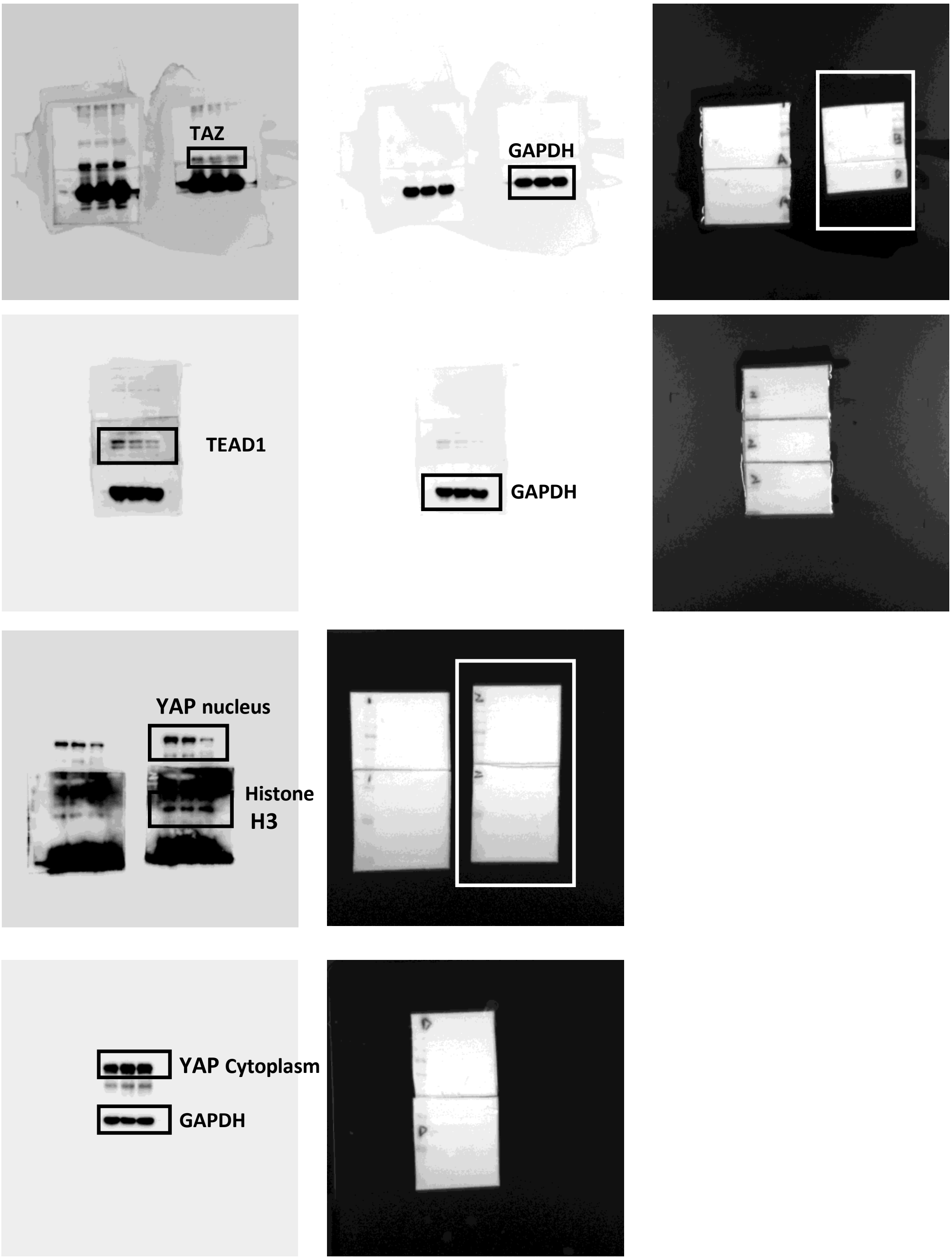

HepG2（Figure 4）

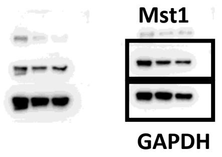

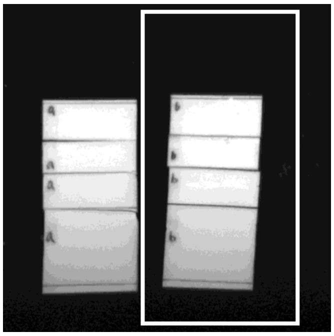

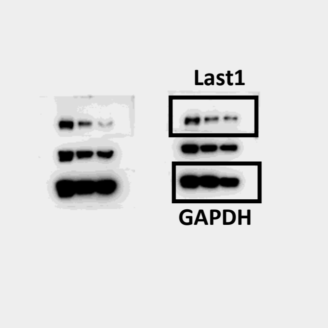

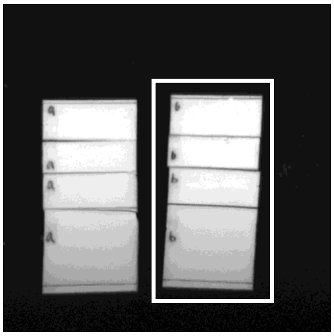

SMMC-7721（Figure4）

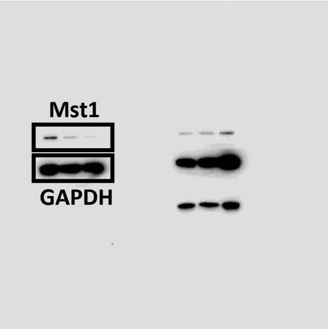

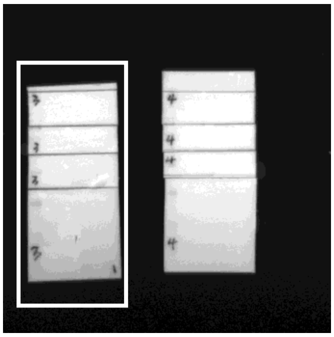

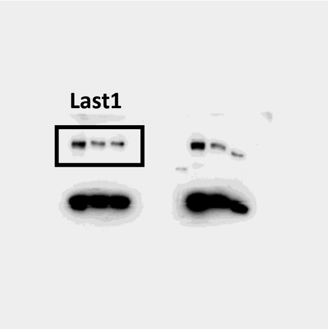

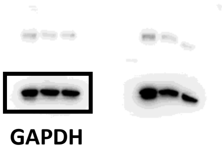

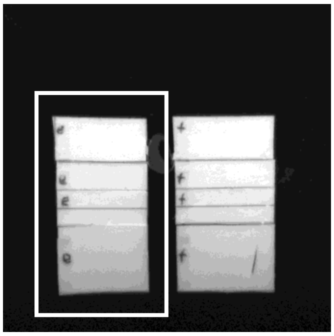

HepG2（Figure 5）

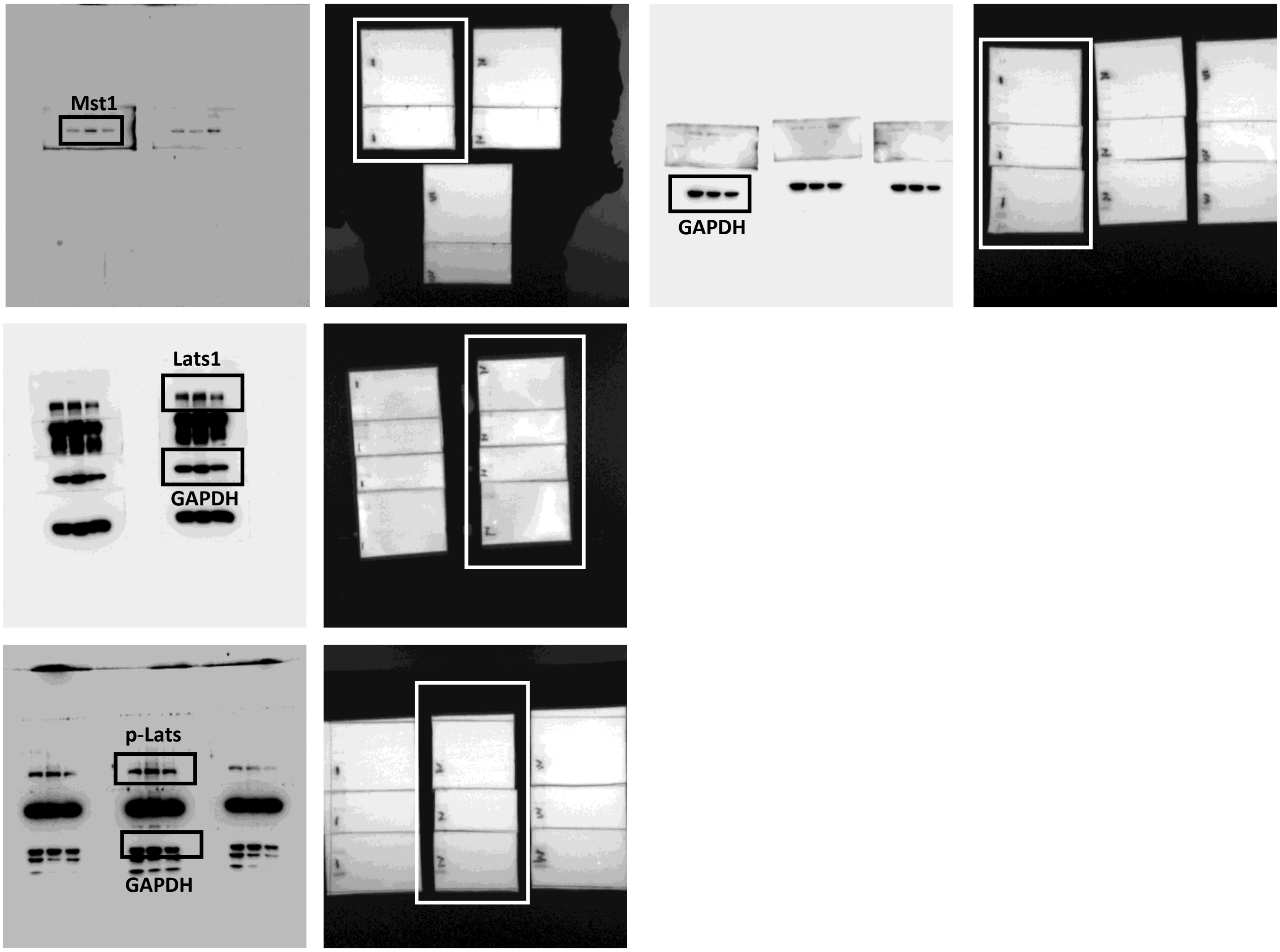

HepG2（Figure 5）

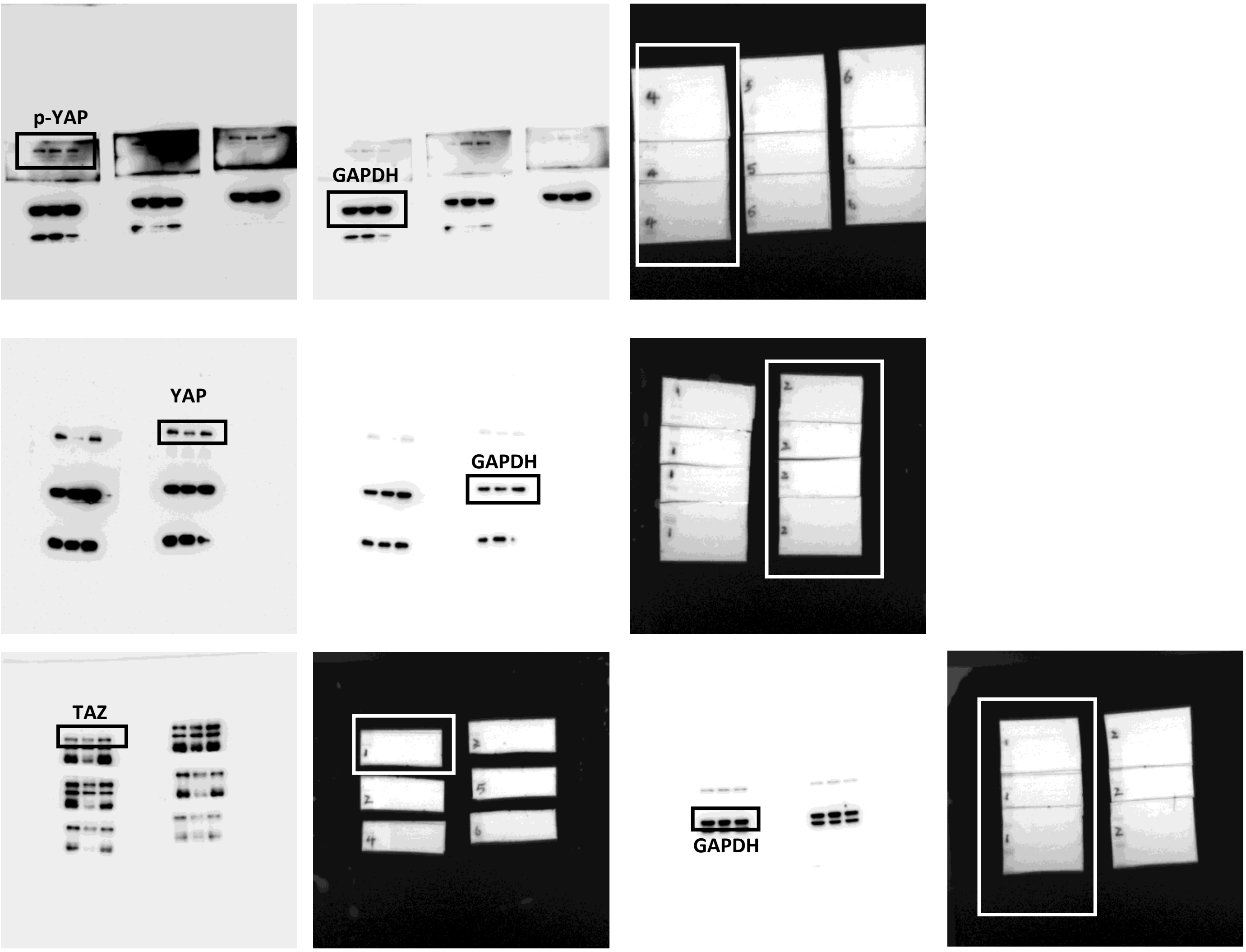

SMMC-7721（Figure 5）

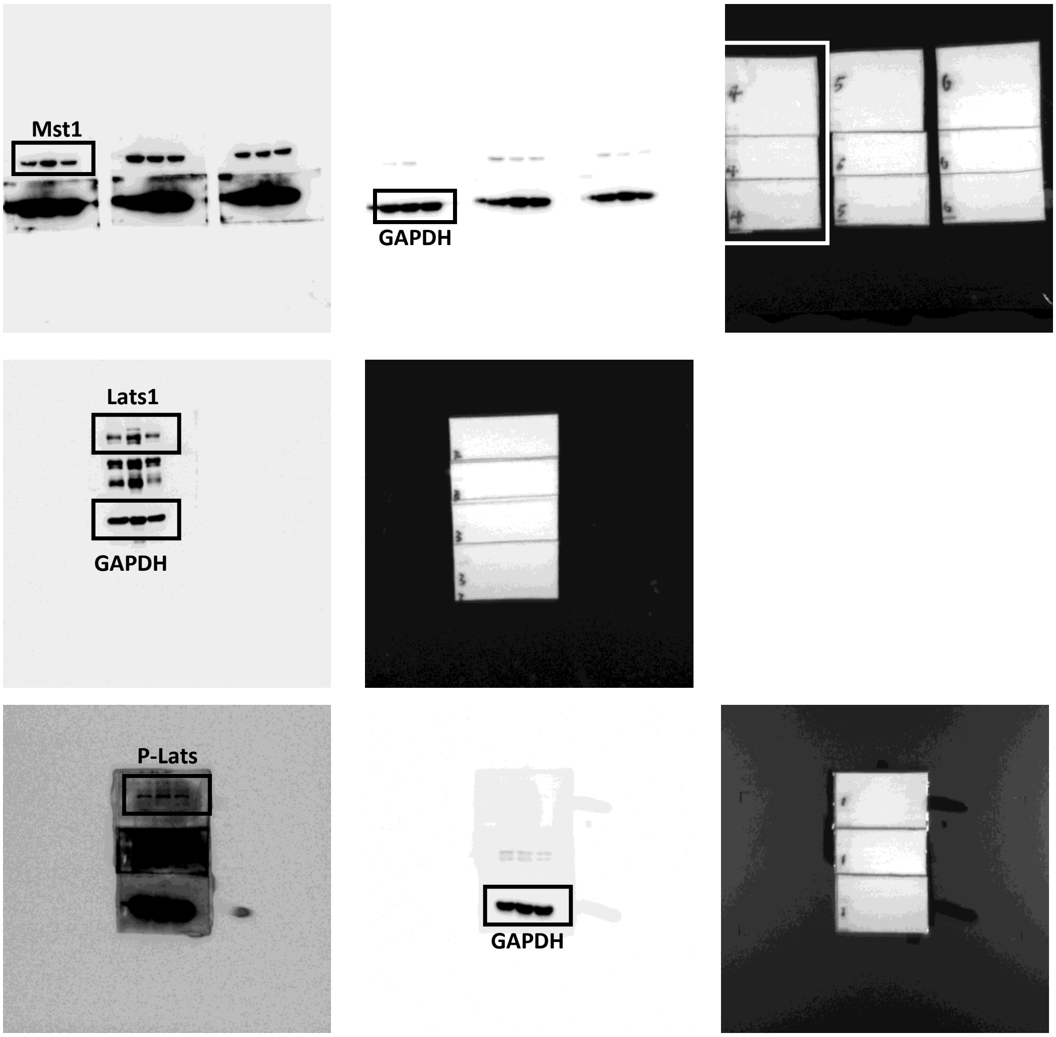

SMMC-7721（Figure 5）

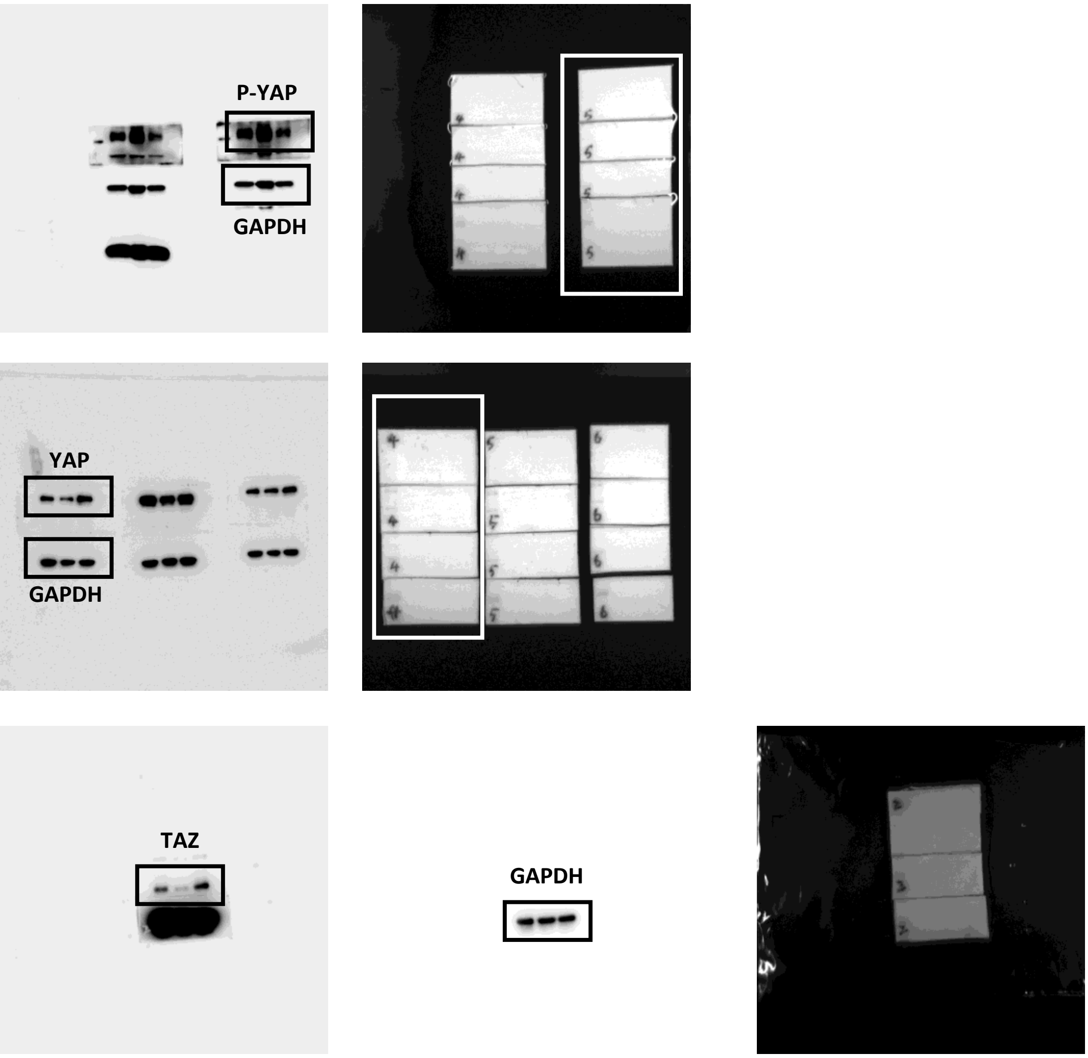

Figure 6

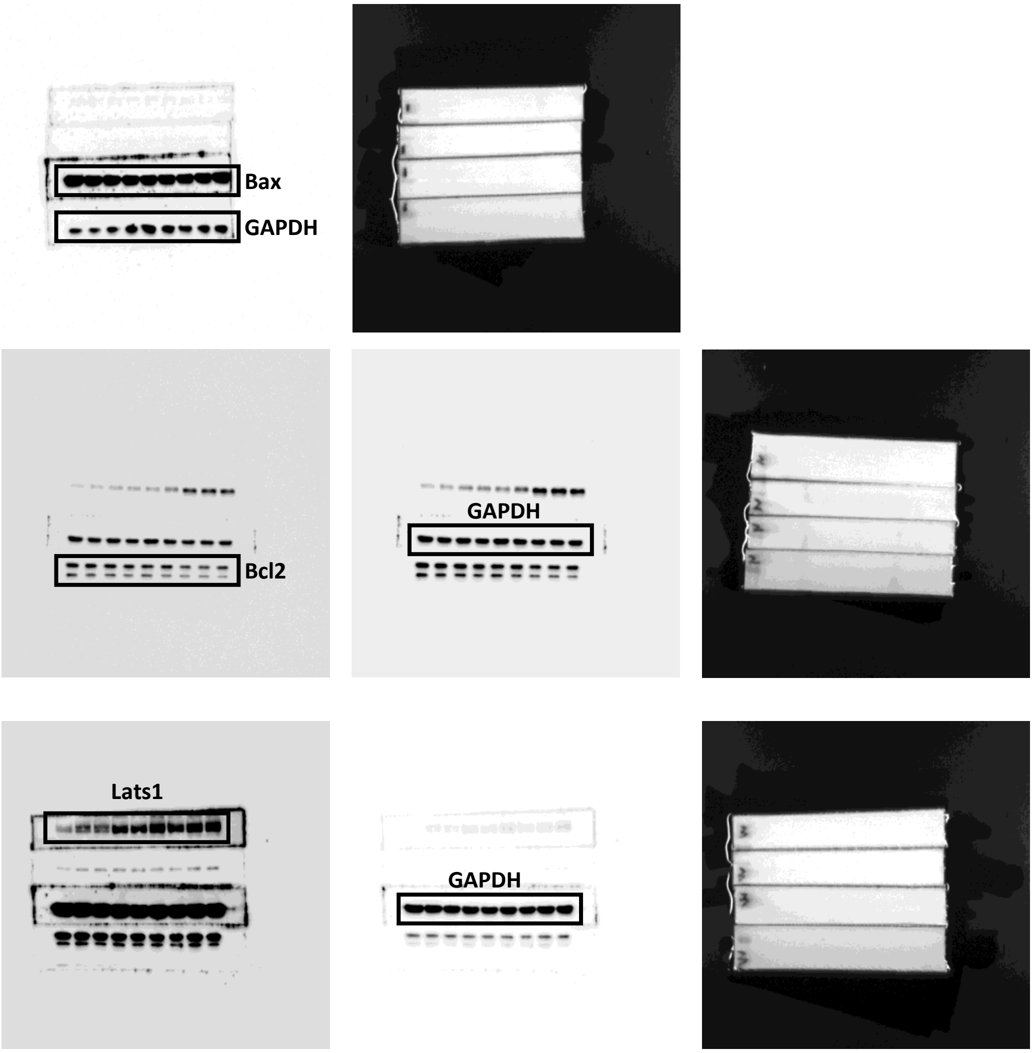

Figure 6

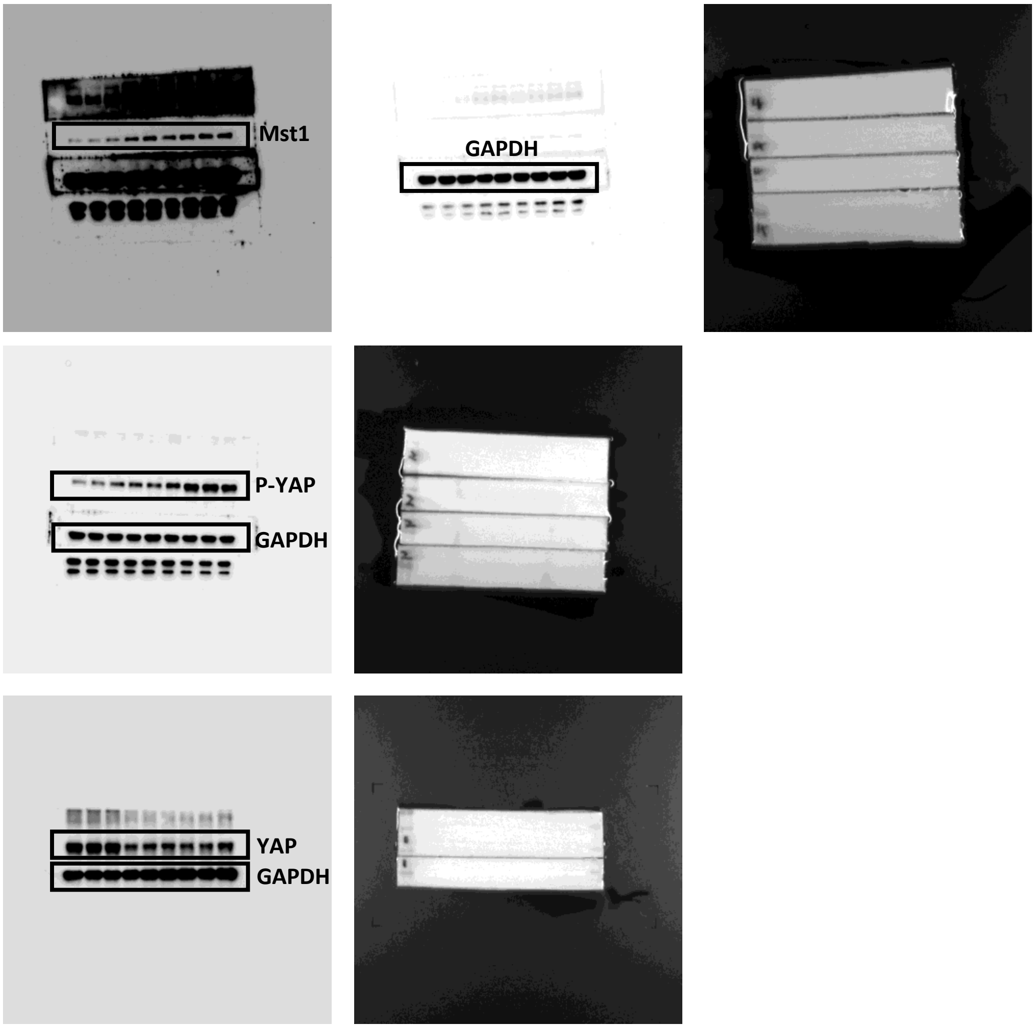

Supplement: Supplementary file 1 [file DataSheet_1.pdf]
